# *In vivo* pharmacokinetics, therapeutic efficacy and immune response of bacteriophage vB_AbaSt_W16 against carbapenem-resistant *Acinetobacter baumannii*

**DOI:** 10.1093/jacamr/dlaf121

**Published:** 2025-07-31

**Authors:** Yoon-Jung Choi, Md Shamsuzzaman, Jae-Eon Lee, Yong Hyun Jeon, Hyungjin Kim, Young-Ran Yoon, Md Shohel Rana, Joohun Shin, Shukho Kim, Jungmin Kim

**Affiliations:** Untreatable Infectious Disease Institute, Kyungpook National University, Daegu, Republic of Korea; Department of Microbiology, School of Medicine, Kyungpook National University, Daegu, Republic of Korea; Department of Microbiology, School of Medicine, Kyungpook National University, Daegu, Republic of Korea; Department of Biomedical Sciences, The Graduate School, Kyungpook National University, Daegu, Republic of Korea; Preclinical Research Centre, Daegu-Gyeongbuk Medical Innovation Foundation (K-MEDIhub), Daegu, Republic of Korea; Preclinical Research Centre, Daegu-Gyeongbuk Medical Innovation Foundation (K-MEDIhub), Daegu, Republic of Korea; School of Medicine, Kyungpook National University, Daegu, Republic of Korea; Department of Clinical Pharmacology and Therapeutics, Kyungpook National University Hospital, Daegu, Republic of Korea; School of Medicine, Kyungpook National University, Daegu, Republic of Korea; Department of Clinical Pharmacology and Therapeutics, Kyungpook National University Hospital, Daegu, Republic of Korea; Department of Microbiology, School of Medicine, Kyungpook National University, Daegu, Republic of Korea; Department of Biomedical Sciences, The Graduate School, Kyungpook National University, Daegu, Republic of Korea; Department of Microbiology, School of Medicine, Kyungpook National University, Daegu, Republic of Korea; Department of Biomedical Sciences, The Graduate School, Kyungpook National University, Daegu, Republic of Korea; Untreatable Infectious Disease Institute, Kyungpook National University, Daegu, Republic of Korea; Department of Microbiology, School of Medicine, Kyungpook National University, Daegu, Republic of Korea; Department of Biomedical Sciences, The Graduate School, Kyungpook National University, Daegu, Republic of Korea; Untreatable Infectious Disease Institute, Kyungpook National University, Daegu, Republic of Korea; Department of Microbiology, School of Medicine, Kyungpook National University, Daegu, Republic of Korea; Department of Biomedical Sciences, The Graduate School, Kyungpook National University, Daegu, Republic of Korea

## Abstract

**Background:**

The increasing prevalence of carbapenem-resistant *Acinetobacter baumannii* (CRAB) infections necessitates alternative therapeutic strategies. Bacteriophage therapy has emerged as a promising approach, yet its clinical implementation is hindered by limited pharmacokinetic (PK) and pharmacodynamic (PD) data.

**Methods:**

The PK and PD properties of *Acinetobacter* phage vB_AbaSt_W16 were evaluated in a murine model. Systemic distribution, clearance kinetics and efficacy were assessed following oral (PO) and intraperitoneal (IP) administration. Conventional PK/PD analysis and real-time fluorescence imaging were used to examine *in vivo* phage dynamics.

**Results:**

After administration, vB_AbaSt_W16 rapidly disseminated systemically within 1 h, reaching peak concentrations at 8 h. Most tissues cleared the phage within 72 h, though residual amounts persisted in the spleen for up to 92 h. In a murine infection model, vB_AbaSt_W16 demonstrated potent antibacterial activity, reducing CRAB bacterial loads by 4–7 log₁₀ cfu/mL within 24 h. Compared with PO administration, IP administration resulted in higher systemic bioavailability and bacterial clearance. Fluorescence imaging enabled non-invasive, real-time monitoring of phage distribution, demonstrating its utility as a PK assessment tool. Notably, phage treatment did not trigger significant pro-inflammatory cytokine release (TNF-α, IL-6) in healthy mice and effectively reduced CRAB-induced inflammation.

**Conclusions:**

These findings highlight the therapeutic potential of vB_AbaSt_W16 and provide critical insights into its PK behaviour. The results support further clinical development of this phage for CRAB infections.

## Introduction

The global rise of antimicrobial resistance (AMR) presents a significant challenge to public health, particularly among Gram-negative bacteria such as *Acinetobacter baumannii*.^1–3^ This opportunistic pathogen is a leading cause of nosocomial infections, including ventilator-associated pneumonia, septicaemia and wound infections, particularly in immunocompromised patients.^1^  *A. baumannii* rapidly acquires resistance through horizontal gene transfer and efflux pumps, reducing antibiotic efficacy.^2,4–6^

The WHO has designated carbapenem-resistant *Acinetobacter baumannii* (CRAB) as one of the highest-priority pathogens requiring urgent action, due to its high mortality rates and limited treatment options, especially in ICU settings.^7^ Given these challenges, bacteriophage (phage) therapy offers a promising alternative for treating MDR bacterial infections.^8–12^

Phages selectively infect and lyse bacteria, offering a highly specific and self-replicating antimicrobial approach.^13^ Unlike broad-spectrum antibiotics, phages minimize disruption of the host microbiota, reducing the risk of dysbiosis and secondary infections.^14,15^ Additionally, phages have demonstrated efficacy in targeting biofilm-associated bacteria, which are often resistant to antibiotic treatment.^16,17^ Despite these advantages, clinical adoption of phage therapy is hindered by rapid immune clearance, bacterial resistance and variable *in vivo* efficacy.^15,18,19^ Strategies such as phage cocktails and combination therapies with antibiotics have been explored to enhance antimicrobial efficacy and mitigate resistance development.^15,20^

A major limitation in the clinical implementation of phage therapy is the lack of comprehensive pharmacokinetic (PK) and pharmacodynamic (PD) data. Unlike conventional antibiotics, phages exhibit self-replicating dynamics that result in non-linear kinetics, complicating traditional PK modelling.^21^ Key factors influencing phage PK/PD include bacterial density, timing of administration and host immune responses, as demonstrated in modelling and *in vivo* studies involving *Escherichia coli*, *Pseudomonas aeruginosa* and *P. aeruginosa* F-8.^14,21^ Effective phage therapy requires bacterial loads to surpass a critical threshold to sustain phage replication; otherwise, premature clearance may limit therapeutic efficacy.^22^ While previous studies have primarily focused on PD aspects, such as bacterial lysis kinetics and host range specificity, research on PK properties—especially for systemically administered phages—remains limited.^18,23^

The *Acinetobacter* phage vB_AbaSt_W16 (GenBank accession no. PP174317) was previously characterized for its strong lytic activity against CRAB.^24^ Initial studies demonstrated its efficacy at various multiplicities of infection (MOI), highlighting its therapeutic potential. Recent research has also explored its synergistic effects when combined with antibiotics such as colistin and meropenem to counteract phage resistance and enhance antimicrobial efficacy (Choi et al., preprint data). However, to fully establish its clinical applicability, a thorough investigation of its PK/PD characteristics is required.

To bridge this gap, we performed a comprehensive preclinical PK/PD study of vB_AbaSt_W16 in a murine model. An MOI of 1 was selected for therapeutic dosing based on unpublished preliminary *in vivo* data comparing MOIs ranging from 0.01 to 1000, in which MOIs of 0.1 and 1 exhibited the most effective bacterial clearance. This dosing strategy reflects clinically relevant phage applications and is currently being evaluated in a separate study investigating phage–antibiotic synergy (PAS) (manuscript under revision). We evaluate its systemic distribution, clearance kinetics and bactericidal efficacy following intraperitoneal (IP) and oral (PO) administration. Additionally, we explore the use of fluorescence imaging as a non-invasive approach for PK analysis, reducing the need for excessive animal sacrifice. By integrating conventional PK/PD analysis with real-time imaging, this study provides critical insights into the therapeutic feasibility of vB_AbaSt_W16 and lays the groundwork for its potential clinical application in treating CRAB infections.

## Materials and methods

### Ethical approval and animal care

This study was conducted in accordance with the Korean Animal Protection Act and was approved by the Institutional Animal Care and Use Committee (IACUC) of the Daegu-Gyeongbuk Advanced Medical Industry Promotion Foundation Preclinical Centre, South Korea (Approval No. KMEDI-24040404-00).

BALB/c mice (6–8 weeks old, 23 ± 2.5 g) were obtained from Goyang Tech (Pyeongtaek, South Korea) and acclimatized for 11 days before experimentation. Mice were housed under controlled conditions (22 ± 2°C, 50 ± 10% humidity, 12 h light/dark cycle) with *ad libitum* access to an antibiotic-free diet and water. Anaesthesia was induced using 2%–2.5% isoflurane vapour, and humane euthanasia was performed via cervical dislocation, adhering to the 3Rs principle (Replacement, Reduction and Refinement).

### Bacterial culture and phage preparation

The CRAB clinical isolate KBN10P02782 was obtained from the Pathogen Resource Bank at Kyungpook National University Hospital, South Korea. The strain was cultured in brain heart infusion (BHI) broth and agar at 37°C and stored at −70°C in 15% glycerol.^24^

Phage vB_AbaSt_W16 was propagated using CRAB KBN10P02782 as its host. The bacterial culture was grown to an optical density at 600 nm (OD_600_) of 0.5 (∼10⁸ cfu/mL), after which the phage was introduced and incubated at 25°C with shaking (200 rpm) for 18 h. The culture was centrifuged (12 000 × **g**, 10 min, 4°C), and the supernatant was filtered through a 0.22 μm syringe filter.

Purification was performed using the Phage on Tap (PoT) method with Amicon Ultra-15 centrifugal filters (10 kDa MWCO, Merck Millipore, Ireland).^25^ The filtrate was further purified via repeated centrifugation in SM buffer (50 mM Tris–HCl, 150 mM NaCl, 10 mM MgCl₂, 2 mM CaCl₂, pH 7.5). Sterility was ensured using 10% chloroform. The final phage stock was adjusted to 1.0 × 10^14^ PFU/mL.

### Thermal and pH stability assays

For thermal stability assessment, 2 mL of vB_AbaSt_W16 (1.0 × 10⁹ PFU/mL) was incubated at temperatures ranging from 4°C to 70°C for 2 h. Viability was evaluated using a double-layer agar (DLA) plaque assay.^26^

For pH stability, phage suspensions were adjusted to pH 2–10 using NaOH or HCl and incubated at 37°C for 2 h. Viable phage titres were determined via plaque assays.^26,27^

### Pharmacokinetics in non-infectious models

Seventy BALB/c mice were divided into three groups:

- Control group (*n* = 10): received 200 μL of SM buffer via PO or IP administration (five mice per route).
- PO phage group (*n* = 30): received 200 μL of vB_AbaSt_W16 (4.85 × 10⁸ PFU/mL) via PO administration.
- IP phage group (*n* = 30): received 200 μL of vB_AbaSt_W16 (4.85 × 10⁸ PFU/mL) via IP injection.

Blood and tissue samples (lungs, liver, spleen, kidneys) were collected at 1, 4, 8, 24, 48 and 72 h post-administration (*n* = 5 per time point). Phage titres were quantified via plaque assays.

### Pharmacokinetics and pharmacodynamics in infection models

A total of 100 BALB/c mice were divided into four groups:

- Control group (*n* = 10): received 200 μL of SM buffer via PO or IP administration (five mice per route).
- Infected control (*n* = 30): infected with *A. baumannii* KBN10P02782 (1.00 × 10⁸ cfu/mL) via IP injection, followed by SM buffer administration.
- Phage-treated (IP, *n* = 30): infected as above and treated with 200 μL of vB_AbaSt_W16 (MOI = 1) via IP injection 30 min after infection.
- Phage-treated (PO, *n* = 30): infected as above and treated with 200 μL of vB_AbaSt_W16 via PO administration 30 min after infection.

Blood and tissue samples were collected at 4, 8, 24, 48, 72 and 96 h post-treatment (*n* = 5 per time point). Bacterial loads were quantified as cfu/mL (blood) or cfu/g (tissue), and phage titres were assessed via plaque assays.

### Fluorescence imaging of pharmacokinetics

#### Phage labelling and preparation

Phage vB_AbaSt_W16 was labelled with FSD Fluor^™^ 647 NHS ester (10 mg/mL in DMSO). Unbound dye was removed using an Amicon Ultracel 10 kDa centrifugal filter (8000 rpm, 15 min, 4°C).

#### *In vivo* and *ex vivo* fluorescence imaging

Prior to imaging, mice were shaved on both dorsal and ventral surfaces to minimize signal interference. They were divided into three groups:

- IP phage group (*n* = 4): received 200 μL of labelled vB_AbaSt_W16 via IP injection.
- PO phage group (*n* = 4): received 200 μL of labelled vB_AbaSt_W16 via PO administration.
- Control group (*n* = 4): received 200 μL of SM buffer via IP administration.

Fluorescence imaging was performed at 1, 4, 8, 24, 48 and 72 h post-administration using the IVIS Spectrum-CT system at the Daegu-Gyeongbuk Advanced Medical Industry Promotion Foundation Preclinical Centre in Daegu, South Korea. Fluorescence intensity was quantified using Living Image software (PerkinElmer, USA) and expressed as total radiant efficiency [(photons/sec)/(µW/cm²)]. These values were analysed to evaluate phage distribution and clearance, and differences in fluorescence intensity over time and between administration routes were assessed to determine the efficiency of systemic circulation and localized phage accumulation. At 72 h, mice were euthanized, and major organs were excised for *ex vivo* imaging.

### Histopathological analysis

Lung tissues were collected 48 h post-treatment, fixed in 10% neutral-buffered formalin and embedded in paraffin. Sections were stained with haematoxylin and eosin (H&E) for histological assessment.

### Cytokine quantification

Blood samples were collected 24 h post-treatment to quantify pro-inflammatory cytokines, including TNF-α and IL-6. Total RNA was extracted from blood samples using the miRNeasy Mini Kit (Qiagen, Hilden, Germany). cDNA synthesis was performed with the ReverTra Ace^™^ qPCR RT Kit (Toyobo, Osaka, Japan), and IL-6 mRNA expression was subsequently analysed using quantitative RT–PCR (qRT–PCR).

qRT–PCR was conducted with SYBR^®^ Green reagent on a real-time PCR system (Applied Biosystems Inc., Foster City, CA, USA). The PCR protocol consisted of an initial denaturation at 95°C for 10 min, followed by 45 cycles of denaturation at 95°C for 15 s, annealing at 55°C for 15 s and extension at 72°C for 10 s. Advanced Relative Quantification analysis was used to validate the expected qRT–PCR product, with gene expression levels normalized to GAPDH using the 2^⁻ΔΔCt^ method. The primer sequences for GAPDH, TNF-α and IL-6 are provided in Table S1 (available as Supplementary data at *JAC-AMR* Online).

### Pharmacokinetic and statistical analysis

#### Pharmacokinetic analysis

PK parameters were analysed using a non-compartmental approach to characterize phage absorption, distribution and clearance profiles. The analysis included maximum plasma concentration (*Cₘₐₓ*), time to reach maximum concentration (*Tₘₐₓ*) and *AUC*, which was computed using the linear trapezoidal rule up to the last quantifiable concentration (*Tₗₐₛₜ*). The elimination half-life (*T*₁/₂) was determined using a one-phase exponential decay model (*T*₁/₂ = 0.693/*Kₑₗ*), where the elimination rate constant (*Kₑₗ*) was derived from the slope of the log-linear phase of the concentration–time curve via least squares regression. All available data, including variability in the elimination phase, were incorporated into the final analysis.

#### Statistical comparisons

Data were expressed as mean ± standard error of the mean (SEM). Group comparisons were performed using one-way ANOVA followed by Tukey’s *post hoc* test to adjust for multiple comparisons. Pairwise comparisons were conducted using Student’s *t*-test. Inter-individual PD variability was assessed and reported as the coefficient of variation (*CV%*). These statistical analyses were conducted to determine whether differences in phage distribution, clearance and immune response between administration routes were statistically significant.

#### Software and significance threshold

Statistical analyses were performed using R v4.2.2 provided by the R Foundation for Statistical Computing (Vienna, Austria), Python v3.9 developed by the Python Software Foundation (Wilmington, DE, USA), GraphPad Prism v10.4.1 from GraphPad Software (San Diego, CA, USA) and WinNonlin v8.3 from Certara (Princeton, NJ, USA).

## Results

### Thermal and pH stability

In the thermal stability assay, vB_AbaSt_W16 maintained infectivity between 4°C and 50°C (Figure S1A). However, phage titres decreased by more than 4-log_10_ PFU at 60°C, and infectivity was nearly lost at 70°C.

In the pH stability assay, vB_AbaSt_W16 remained stable between pH 4.0 and 9.0 (Figure S1B). At pH 3.0, phage titres declined by approximately 2-log_10_ PFU, while at extreme pH values (pH 2.0 and 10.0), titres dropped by more than 3-log_10_ PFU, indicating significant infectivity loss under highly acidic or alkaline conditions.

### Systemic distribution in non-infected mice

**Figure 1.**
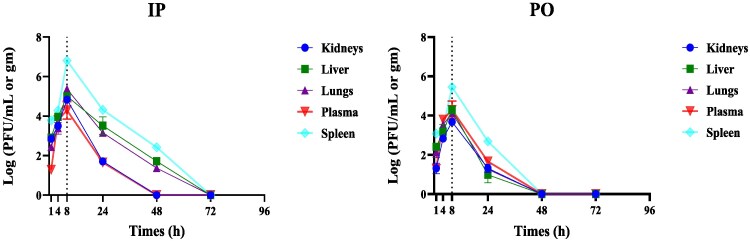
Pharmacokinetics of vB_AbaSt_W16 in non-infected mice. Phage titres (log₁₀ PFU/mL) in blood, lungs, liver, spleen and kidneys were measured at 1, 4, 8, 24, 48 and 72 h post-administration via IP (a) and PO (b) routes. Mice received 200 μL of vB_AbaSt_W16 (4.85 × 10⁸ PFU/mL). Dashed vertical lines represent *Tₘₐₓ* (time to peak phage concentration). Each data point represents the mean ± SEM from three independent experiments (*n* = 5 per time point).

As illustrated in Figure 1 and Table 1, vB_AbaSt_W16 reached *Tₘₐₓ* at 8 h post-administration in non-infected mice. IP administration resulted in significantly higher *Cₘₐₓ* compared with PO administration. The highest phage titres were detected in the spleen (IP: log₁₀ 6.8 PFU/mL; PO: log₁₀ 5.5 PFU/mL), followed by the lungs (IP: log₁₀ 5.4; PO: log₁₀ 4.7), liver (IP: log₁₀ 5.0; PO: log₁₀ 4.3), kidneys (IP: log₁₀ 4.8; PO: log₁₀ 3.7) and blood (IP: log₁₀ 4.5; PO: log₁₀ 3.7) (*P* < 0.0001). The elimination half-life (*T*₁/₂) varied across tissues, with the longest observed in the spleen (IP: 26.82 h; PO: 21.90 h), suggesting prolonged phage retention. The shortest *T*₁/₂ was observed in the blood (IP: 11.65 h; PO: 9.30 h), indicating rapid clearance.

**Table 1.** Estimated PK parameters of *Acinetobacter* phage vB_AbaSt_W16

Organ	Blood		Kidneys		Liver		Lungs		Spleen	
Route of administration	IP	PO	IP	PO	IP	PO	IP	PO	IP	PO
Parameters	Administered dose: 200 µL of 4.85 × 10^8^ PFU/mL of vB_AbaSt_W16									
*C_max_* (PFU/mL)	34 800	5160	69 400	5160	105 600	21 800	236 000	13 340	6 500 000	288 200
*T_max_* (h)	8	8	8	8	8	8	8	8	8	8
*V_d_* (mL)	1537	6674	314	6246	541	2186	238	8120	8	608
*T*_1/2_ (h)	11.65	9.30	13.65	10.78	25.87	18.75	20.30	23.38	26.82	21.90
*CL* (mL/h)	261.29	1830.57	137.05	1779.15	80.70	374.18	40.41	594.17	1.48	32.96
*AUC_0_–t* (PFU ∗ h/mL)	371 235	52 989	707 780	54 520	1 201 945	259 234	2 400 133	163 253	65 519 524	2 942 968
Relative bioavailability (%)	14.16		7.67		21.57		6.81		4.48	

In the presence of host bacteria										
Organ	Blood		Kidneys		Liver		Lungs		Spleen	
Route of administration	IP	Oral	IP	Oral	IP	Oral	IP	Oral	IP	Oral
Parameters	Administered dose: 200 µL of 1.0 × 10^8^ cfu/mL of *A. baumannii* KBN10P02782 + 200 µL of 4.85 × 10^8^ PFU/mL of vB_AbaSt_W16									
*C_max_* (PFU/mL)	23 920 000	191 000	71 600 000	610 800	2 062 000 000	1 798 000	13 560 000 000	25 520 000	231 600 000 000	488 000 000
*T_max_* (h)	24	24	24	24	24	24	24	24	24	24
*V_d_* (mL)	535.08	60 010.72	219.61	27 622.98	16.79	6729.32	1.45	645.25	0.09	36.64
*T*_1/2_ (h)	46.59	40.05	51.32	45.24	52.66	42.76	58.13	47.22	73.67	52.91
*CL* (mL/h)	199.11	22 696.81	69.83	8416.06	5.59	2582.47	0.36	193.32	0.02	10.57
*AUC_0_–t* (PFU ∗ h/mL)	487 553 504	4 277 900	1 447 442 430	12 356 210	42 997 589 686	37 719 390	273 908 474 020	510 734 071	4 701 465 004 728	9 765 399 638
Relative bioavailability (%)	0.88		0.85		0.09		0.19		0.21	

Key PK parameters were estimated for IP and PO administration routes, including maximum plasma concentration (*C_max_*), time to maximum concentration (*T_max_*), volume of distribution (*V_d_*), elimination half-life (*T*₁/₂), clearance (*CL*) and area under the concentration–time curve (*AUC_0_–t*). Relative bioavailability (*F*) was calculated using the formula: *F*, *AUC_0_−t* (PO)/*AUC_0_*−*t* (IP) × 100.

### *In vivo* fluorescence imaging

**Figure 2.**
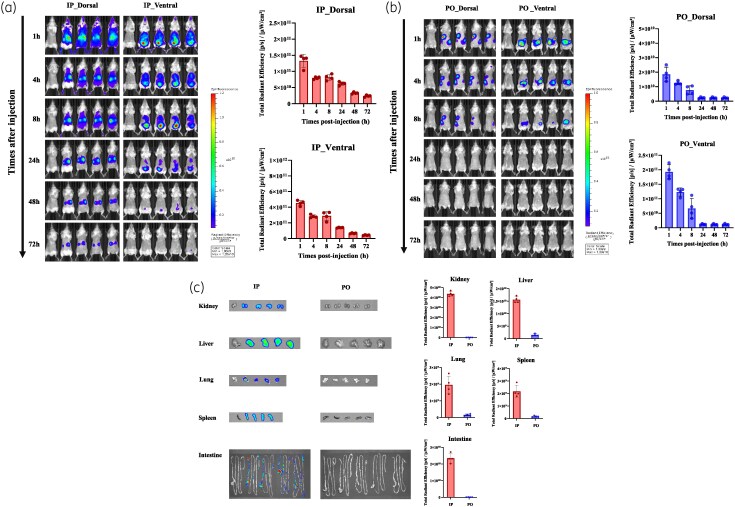
*In vivo* and *ex vivo* fluorescence imaging of vB_AbaSt_W16 in non-infected mice. (a, b) *In vivo* fluorescence imaging of vB_AbaSt_W16 following IP (a) and PO (b) administration in live mice. Mice were administered with 200 μL of FSD Fluor^™^ 647-labelled vB_AbaSt_W16 (1 × 10⁸ PFU/mL), and fluorescence images were acquired at 1, 4, 8, 24, 48 and 72 h using the IVIS Spectrum-CT system. Fluorescence intensity was quantified as total radiant efficiency [(photons/s)/(μW/cm²)]. (c) *Ex vivo* fluorescence imaging of major organs (liver, kidney, lung, spleen and intestines) at 72 h post-administration. Each data point represents the mean ± SEM from three independent experiments (*n* = 4 per time point). Statistical significance was determined using two-way ANOVA (*P* 0.001).

Real-time fluorescence imaging confirmed systemic phage dissemination. IP administration resulted in higher fluorescence intensity than PO administration at all time points (Figure 2a and b). *Ex vivo* imaging at 72 h revealed significantly higher fluorescence signals in the spleen, liver and kidneys of IP-treated mice, whereas PO administration primarily localized to the gastrointestinal tract (Figure 2). Quantitative analysis of *ex vivo* fluorescence signals (Figure 2c) showed that IP administration resulted in significantly higher total radiant efficiency in the spleen, liver, kidneys and lungs compared to PO administration. In contrast, fluorescence signals in the intestines were higher in PO-treated mice, consistent with localized gastrointestinal accumulation.

### Replication and bactericidal activity in infected mice

**Figure 3.**
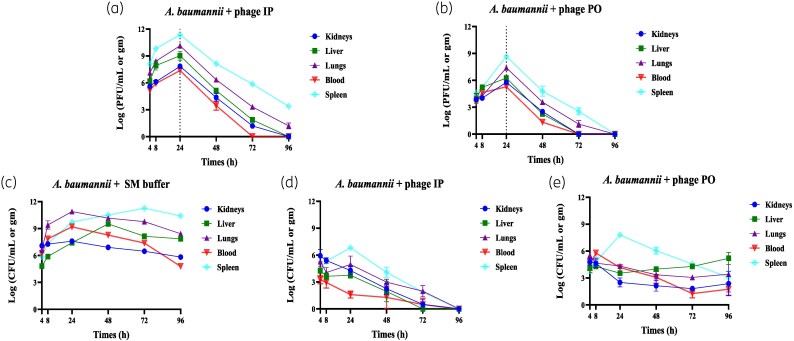
Pharmacokinetics and pharmacodynamics of vB_AbaSt_W16 in CRAB-infected mice. (a, b) Phage titres (log₁₀ PFU/mL) were measured in blood, lungs, liver, spleen and kidneys at 4, 8, 24, 48, 72 and 96 h post-infection following IP (a) and PO (b) administration. Mice were infected with *A. baumannii* KBN10P02782 (1.00 × 10⁸ cfu/mL) via IP injection and treated with 200 μL of vB_AbaSt_W16 (4.85 × 10⁸ PFU/mL). Dashed vertical lines indicate *Tₘₐₓ*. (c–e) Bacterial burden analysis in infected mice: (c) infection control group (*A. baumannii* + SM buffer), (d) IP phage-treated group, (e) PO phage-treated group. Each data point represents the mean ± SEM from three independent experiments (*n* = 5 per time point).

In infected mice, vB_AbaSt_W16 exhibited delayed peak concentrations, reaching *Tₘₐₓ* at 24 h post-treatment (Figure 3a and b; Table 1). *Cₘₐₓ* values were significantly higher than in non-infected mice, particularly in the spleen and lungs, indicating phage replication within bacterial hosts. The *T*₁/₂ was prolonged in infected tissues (IP: 73.67 h; PO: 52.91 h in the spleen), while clearance from circulation was faster (IP: 46.59 h; PO: 40.05 h).

vB_AbaSt_W16 demonstrated potent antibacterial activity against *A. baumannii* KBN10P02782 (Figure 3c–e). Bacterial counts in untreated mice increased over time, peaking at 24–48 h. In contrast, phage-treated mice exhibited a significant bacterial reduction, with a 4–7 log₁₀ decrease in cfu/mL or cfu/g by 72 h (*P* < 0.001).

IP administration resulted in faster bacterial clearance, with undetectable levels achieved in most organs by 48 h. In the PO-treated group, residual bacteria persisted in the spleen beyond 48 h, suggesting a less efficient systemic effect.

### Anti-inflammatory effects of phage treatment

**Figure 4.**
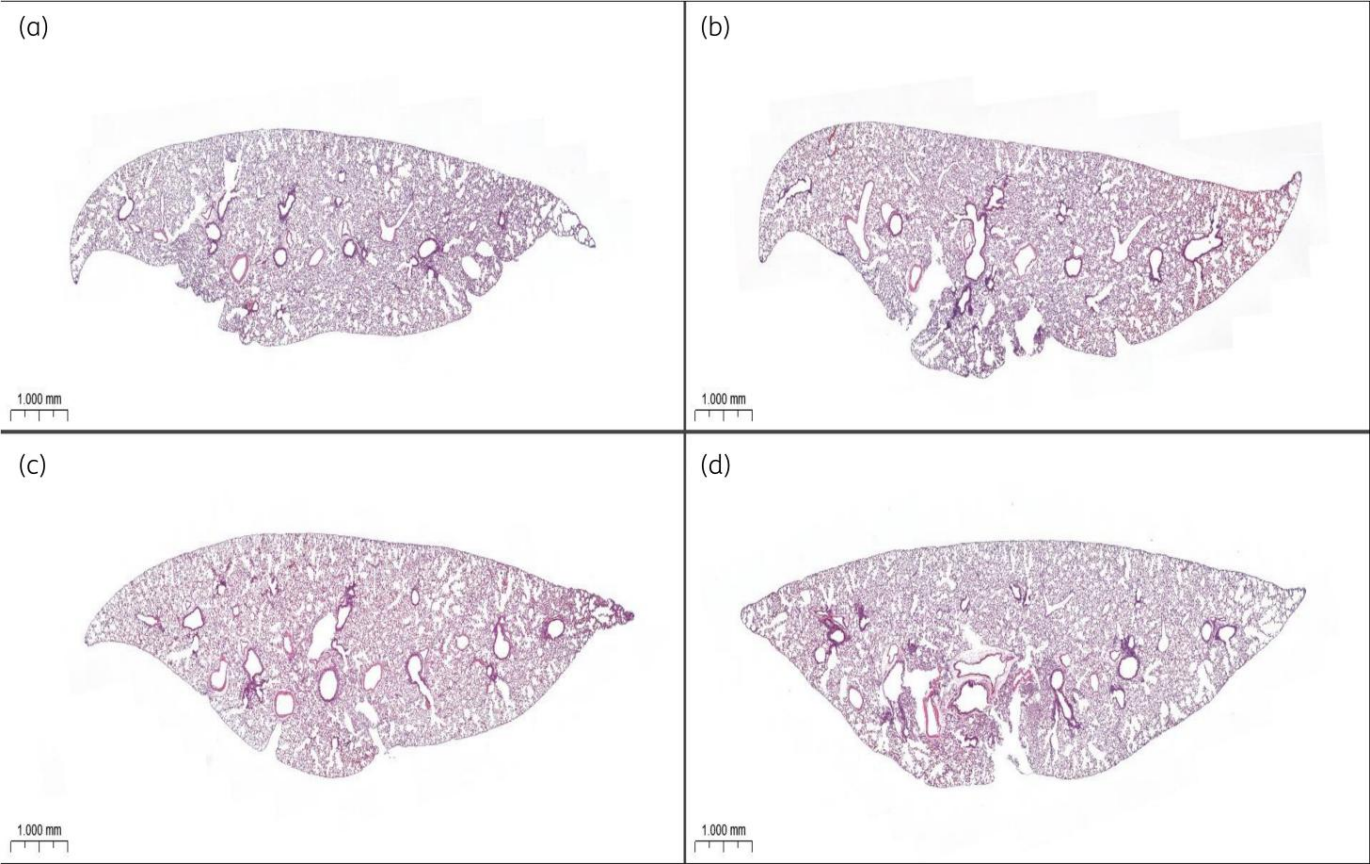
Histopathological analysis of mouse lung tissue. Representative H&E-stained lung tissue sections (20× magnification) from (a) a healthy control mouse; (b) a CRAB-infected untreated mouse, showing severe inflammatory infiltration and alveolar damage; (c) an IP-administered phage-treated mouse, demonstrating reduced inflammation and preserved lung architecture; and (d) a PO-administered phage-treated mouse, showing moderate structural improvement compared with the untreated group.

H&E staining of lung tissues at 48 h post-infection revealed severe inflammatory infiltration, alveolar destruction and tissue damage in untreated infected mice (Figure 4; Figures S2–S4). In contrast, phage-treated mice exhibited significantly reduced inflammation and preserved lung architecture. The IP-treated group demonstrated greater structural preservation compared with the PO group.

**Figure 5.**
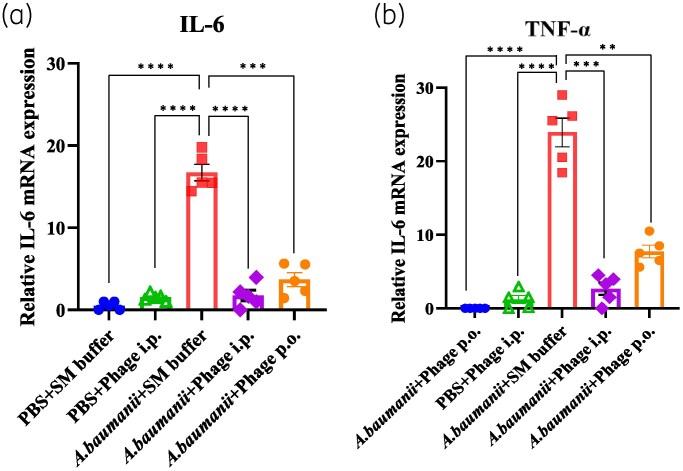
Pro-inflammatory cytokine levels in plasma of infected and treated mice. (a, b) mRNA expression levels of IL-6 (a) and TNF-α (b) were quantified in plasma at 24 h post-infection. Cytokine levels were significantly elevated in untreated CRAB-infected mice compared with uninfected and phage-treated groups (*P *< 0.05). Minimal changes in TNF-α and IL-6 mRNA levels were observed in the vehicle control group (SM buffer). Cytokine mRNA levels were normalized to GAPDH mRNA and expressed as fold-change (2^⁻ΔΔCt^) relative to control groups. Data represent the mean ± SEM from triplicate experiments (*P < 0.05, **P < 0.01, ***P < 0.001, ****P < 0.0001, two-way ANOVA).

As presented in Figure 5, pro-inflammatory cytokines (IL-6 and TNF-α) were significantly elevated in untreated infected mice compared with controls (*P* < 0.0001). Phage treatment resulted in a substantial cytokine reduction, with IL-6 levels decreasing 9.1-fold in the IP group and 4.3-fold in the PO group, and TNF-α levels decreasing 8.5-fold and 3.2-fold, respectively (*P* < 0.05). These findings suggest that vB_AbaSt_W16 effectively reduces CRAB-induced inflammation.

## Discussion

This study presents a comprehensive preclinical evaluation of the PK/PD properties of *Acinetobacter* phage vB_AbaSt_W16, highlighting its potential as a therapeutic option for CRAB.

Our findings indicate that vB_AbaSt_W16 rapidly disseminates systemically, with *Tₘₐₓ* occurring within 8 h in non-infected mice and 24 h in infected mice (Table 1; Figure 1). The delayed peak in infected mice indicates *in vivo* phage replication, consistent with previous studies.^18,21^ IP administration resulted in significantly higher phage concentrations in the bloodstream and tissues compared with PO administration, highlighting its superior systemic bioavailability.

The *T*₁/₂ varied across tissues, with prolonged retention in the spleen, suggesting that this organ may act as a phage reservoir (Table 1). In contrast, rapid clearance of phages from the blood and kidneys was observed, likely mediated by immune clearance via the mononuclear phagocyte system (MPS) in blood and efficient renal filtration. Interestingly, the bioavailability of orally administered phages was significantly lower, with an *F* of only 24% in non-infected mice and less than 1% in infected mice. This suggests that gastrointestinal barriers and immune responses may limit systemic absorption, underscoring the need for optimized oral formulations, such as encapsulation or enteric coatings, to improve stability and bioavailability.^11,28^ Furthermore, although IP administration was more effective in this study, oral delivery remains a preferable route in clinical settings due to its convenience and non-invasiveness. To enhance systemic phage bioavailability via PO administration, additional formulation strategies such as mucoadhesive carriers or pH-sensitive nanoparticles should be considered. These approaches may protect phages during gastrointestinal transit and promote uptake across mucosal barriers.

vB_AbaSt_W16 exhibited strong antibacterial activity, reducing *A. baumannii* bacterial loads by 4–7 log₁₀ cfu/mL within 24 h. IP administration achieved rapid bacterial clearance, with undetectable levels in most tissues by 48 h. In contrast, PO administration resulted in slower clearance, with residual bacteria persisting in some tissues beyond 48 h. This finding suggests that while oral phage therapy may be beneficial for localized gastrointestinal infections, systemic infections may require alternative administration routes for optimal efficacy.^29,30^

The observed efficacy aligns with previous research demonstrating the potential of lytic phages in reducing bacterial burden. Importantly, no significant resistance was detected during the study period, supporting the use of vB_AbaSt_W16 as a viable therapeutic candidate. However, prolonged phage exposure has been associated with the emergence of bacterial resistance.^31,32^ In our study, regrowth of *A. baumannii* was observed at 96 h in the PO-treated group, despite initial bacterial reduction. This observation may indicate the emergence of phage-resistant subpopulations *in vivo*, warranting further investigation. To verify this, we plan to test the phage susceptibility of *A. baumannii* isolates recovered at 96 h post-treatment in future studies. Strategies such as phage–antibiotic combination therapy or phage cocktail formulations may help mitigate resistance development and enhance long-term efficacy.

A major concern in phage therapy is its potential to trigger an immune response that could limit efficacy or cause adverse effects. Our results demonstrate that vB_AbaSt_W16 did not induce significant pro-inflammatory cytokine responses (TNF-α, IL-6) in healthy mice, suggesting a favourable immunological profile. Furthermore, phage treatment significantly reduced inflammation in infected mice, as evidenced by lower cytokine levels and improved lung histopathology. While anti-inflammatory cytokines such as IL-10 were not evaluated in this study, future work will include such analyses to provide a more comprehensive view of immune modulation during phage therapy. These findings support the notion that phage therapy may not only target bacterial infections but also modulate host immune responses to alleviate infection-induced inflammation.^11,33^

Despite these promising results, further studies are needed to evaluate long-term immune interactions, particularly in immunocompromised patients. Future research should assess the impact of neutralizing antibodies on repeated phage dosing and efficacy.

This study highlights the utility of fluorescence imaging as a non-invasive method for real-time PK analysis of phages. Compared with traditional PK assessments that rely on invasive sampling, fluorescence imaging allows dynamic monitoring of phage distribution with minimal animal sacrifice. Our results demonstrate that fluorescence imaging effectively tracked phage dissemination and clearance, providing insights into organ-specific accumulation patterns. However, fluorescence imaging has limitations, including potential signal interference from tissue autofluorescence and the inability to differentiate between viable and inactive phages. Future studies could explore near-infrared (NIR) dyes or reporter gene-tagged phages to improve tracking resolution and distinguish active phage populations.^30^ For example, reporter phages can be engineered to express fluorescent or bioluminescent proteins only upon successful infection of host bacteria, enabling real-time visualization of phage activity rather than mere presence. Additionally, NIR dyes with deeper tissue penetration and lower background autofluorescence could enhance the accuracy of *in vivo* imaging by distinguishing viable phages from degraded or inactivated particles.

Our findings emphasize the therapeutic potential of vB_AbaSt_W16 for treating CRAB infections. However, to maximize clinical applicability, several factors require further investigation, such as optimization of phage formulation, particularly improving oral bioavailability, PAS, large-animal and clinical trials and mathematical (or population) PK/PD modelling.^32,34^

Despite these promising findings, this study has several limitations. The murine model may not fully replicate human infection dynamics, necessitating further validation in larger animal models and clinical trials. Additionally, potential interactions between phages and the host immune system over extended treatment periods require further investigation.

In conclusion, this study provides critical insights into the PK/PD characteristics of vB_AbaSt_W16, demonstrating its potential as an effective phage therapy candidate against CRAB infections. The prolonged tissue persistence, potent bactericidal activity and favourable immunological profile support its therapeutic feasibility. These findings contribute to the growing body of evidence supporting phage therapy as a viable alternative for combating MDR bacterial infections.

## Supplementary Material

dlaf121_Supplementary_Data

## Data Availability

The data supporting the findings of this study have been deposited in the Figshare open-access repository and are publicly available via the following DOI: https://doi.org/10.6084/m9.figshare.29334839.

## Supplementary data

Figures S1–S4 and Table S1 are available as Supplementary data at *JAC-AMR* Online.
