# Supplementary material for: In vivo pharmacokinetics, therapeutic efficacy and immune response of bacteriophage vB_AbaSt_W16 against carbapenem-resistant Acinetobacter baumannii
Source: JAC Antimicrob Resist. 2025 Jul 31;7(4):dlaf121. doi: 10.1093/jacamr/dlaf121 (PMC12310330; doi:10.1093/jacamr/dlaf121)
Supplement: dlaf121_Supplementary_Data [file dlaf121_supplementary_data.zip › Suppl_Table.docx]

| Cytokine | Primer sequences |
| --- | --- |
| IL-6 Forward | 5′-GAGGATACCACTCCCAACAGACC-3′ |
| IL-6 Reverse | 5′-AAGTGCATCATCGTTGTTCATACA-3′ |
| TNF-α Forward | 5′-CATCTTCTCAAAATTCGAGTGACAA-3′ |
| TNF-α Reverse | 5′-TGGGAGTAGACAAGGTACAACCC-3′ |
| β-actin Forward | 5’-CTGTCCCTGTATGCCTCTG-3’ |
| β-actin Reverse | 5’-ATGTCACGCACGATTTCC-3’ |

**Supplementary Table 1. Primer sequences used for qPCR analysis of cytokine expression.**
