# Supplementary figures and images for: In vivo pharmacokinetics, therapeutic efficacy and immune response of bacteriophage vB_AbaSt_W16 against carbapenem-resistant Acinetobacter baumannii
Source: JAC Antimicrob Resist. 2025 Jul 31;7(4):dlaf121. doi: 10.1093/jacamr/dlaf121 (PMC12310330; doi:10.1093/jacamr/dlaf121)

**Supplementary Figure 1.**

**(A)**

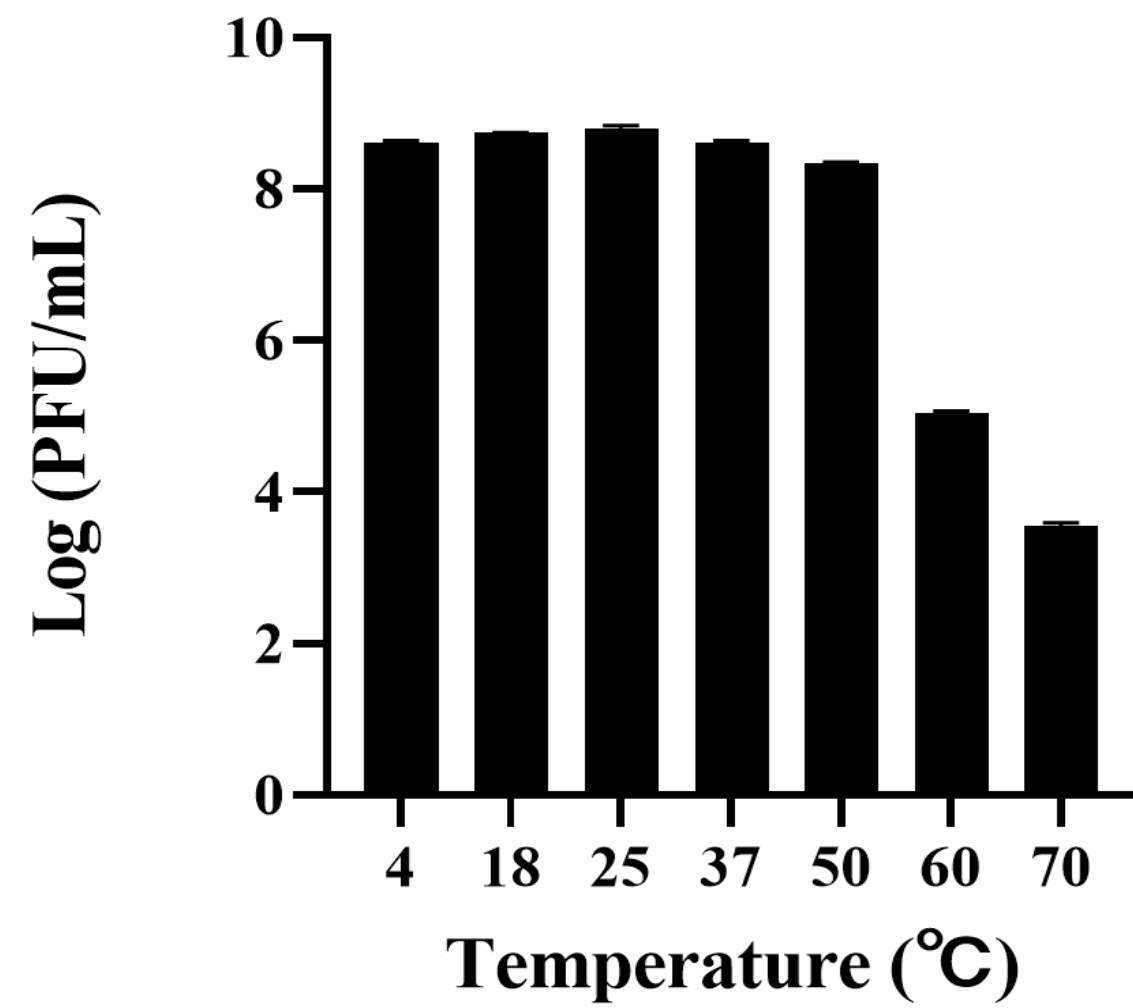

**(B)**

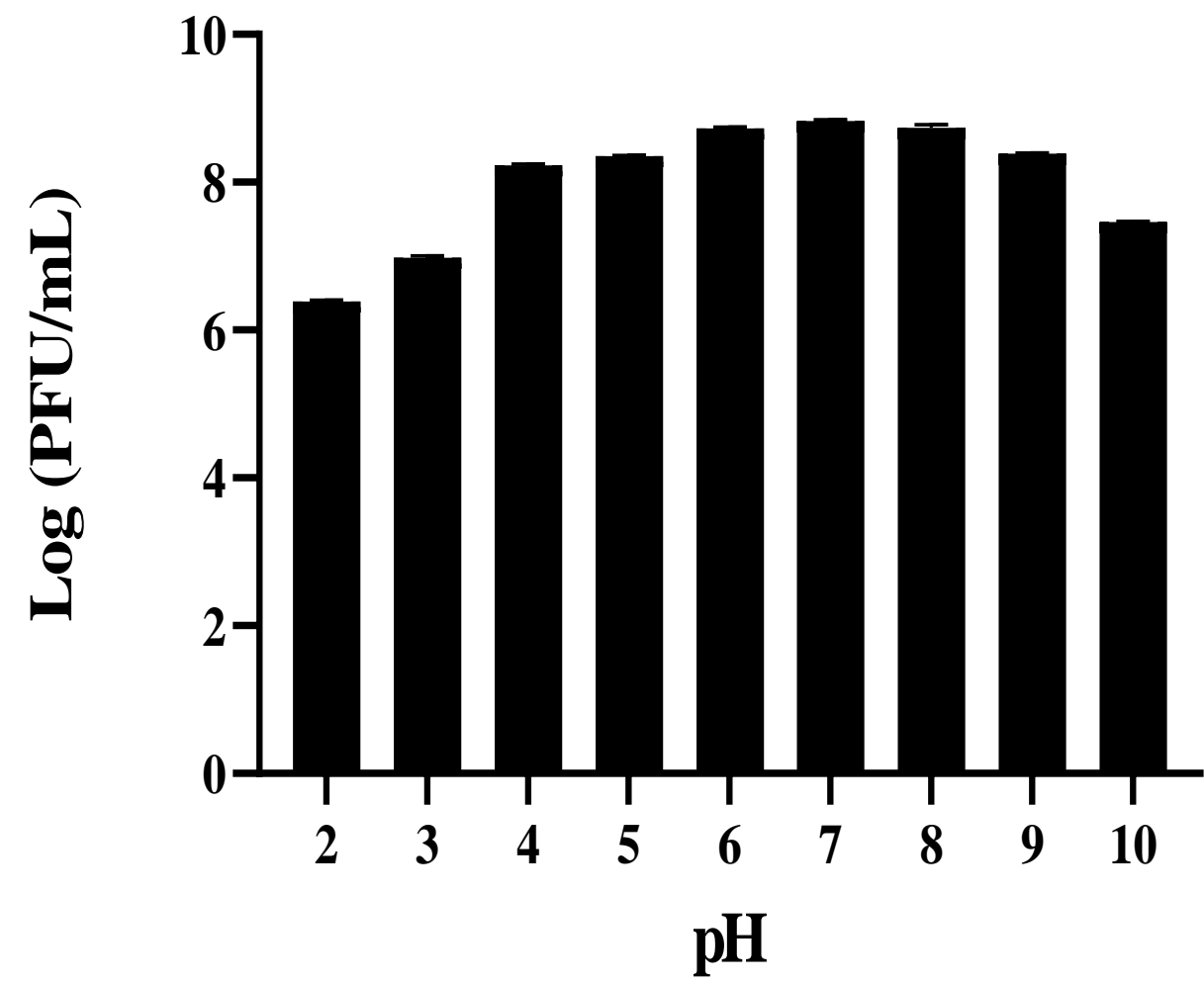

Supplementary Figure 2.

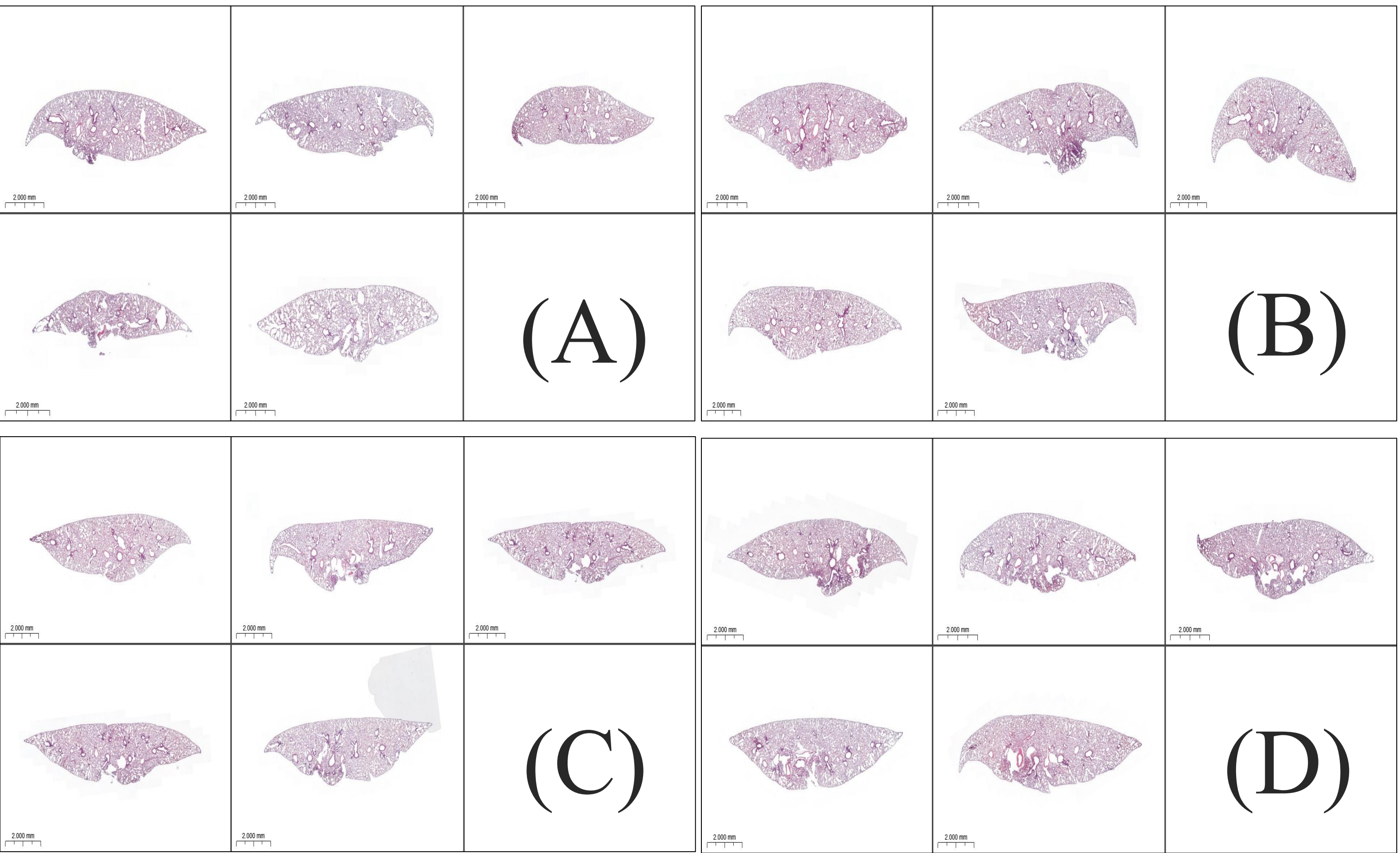

Supplementary Figure 3.

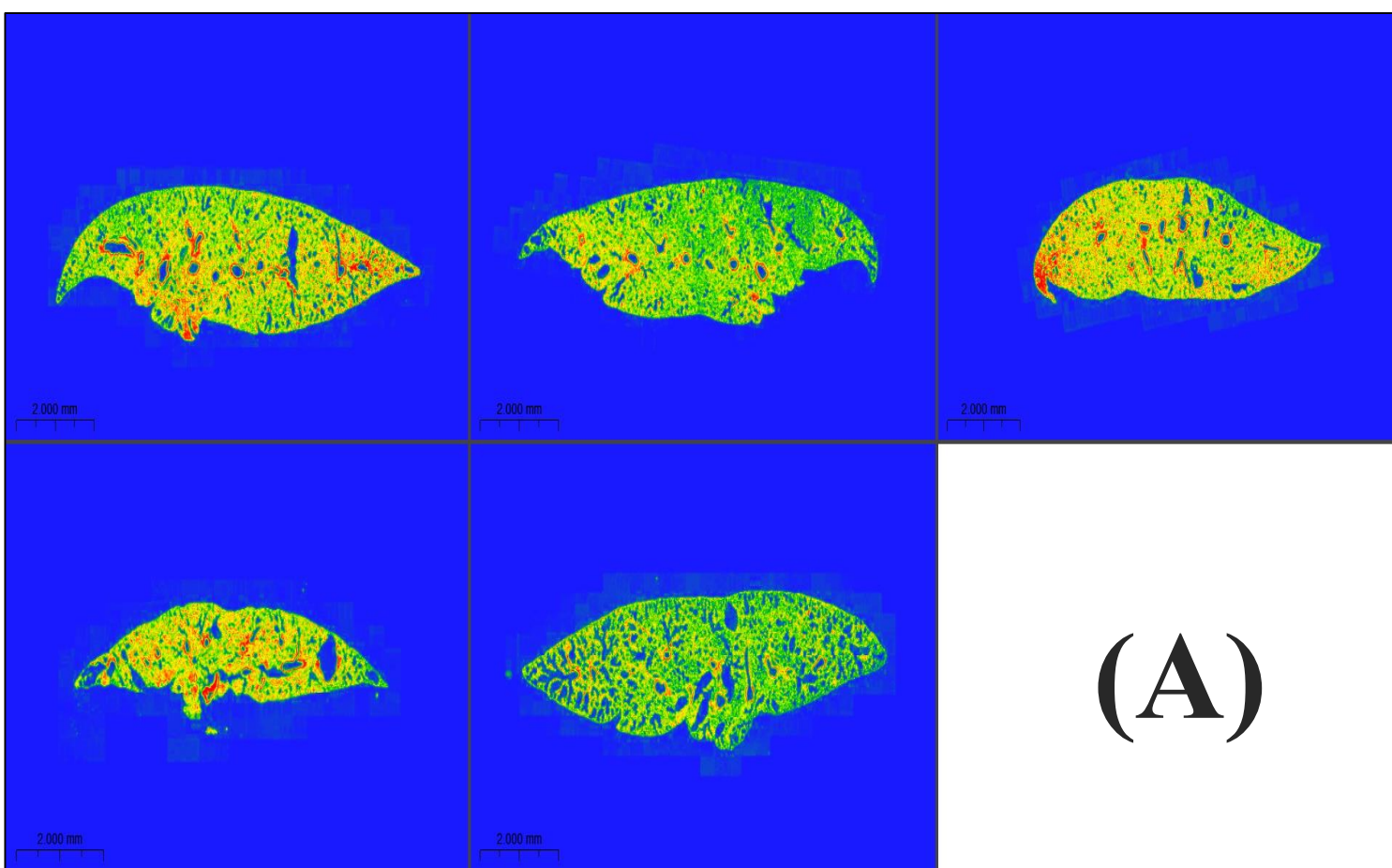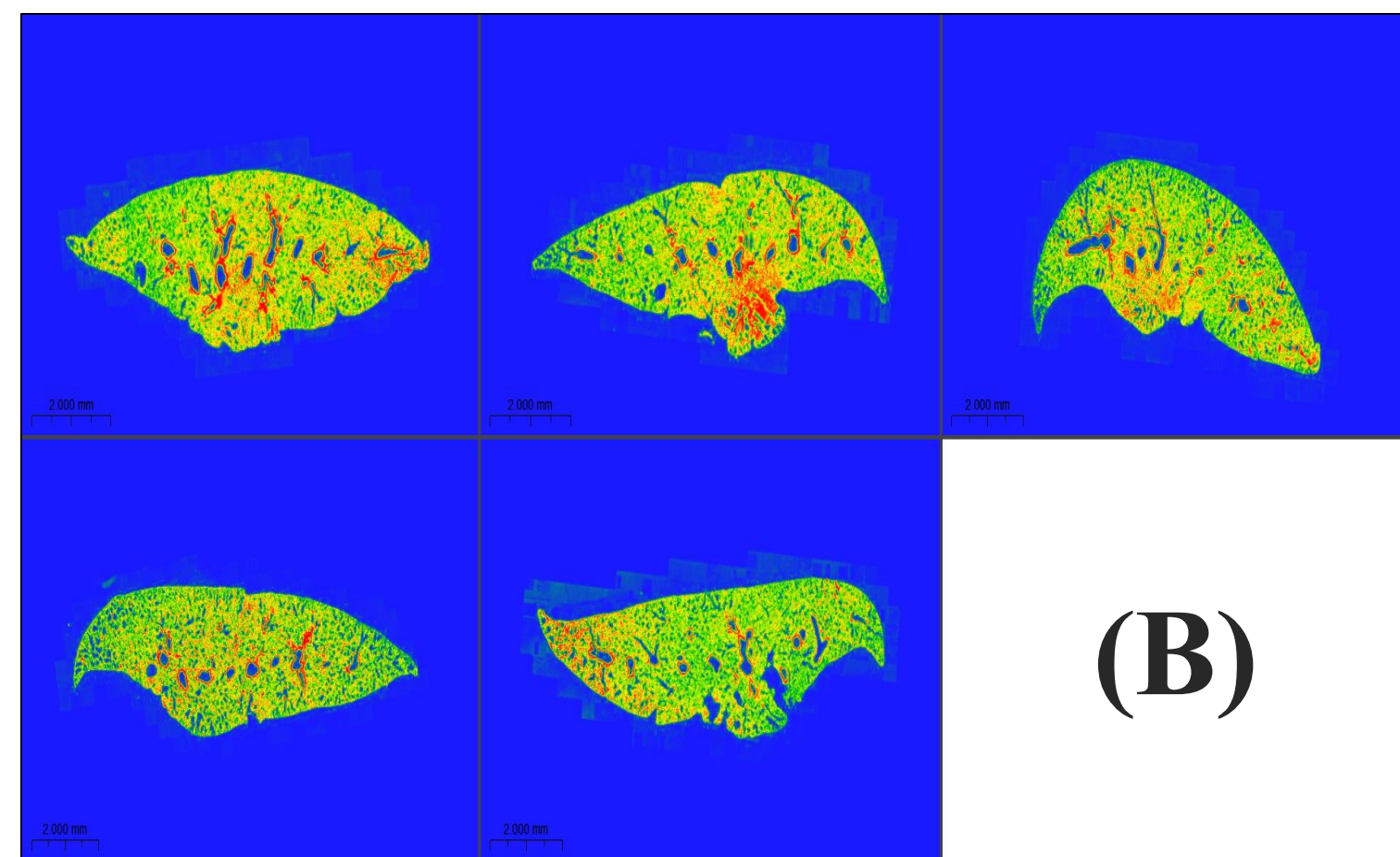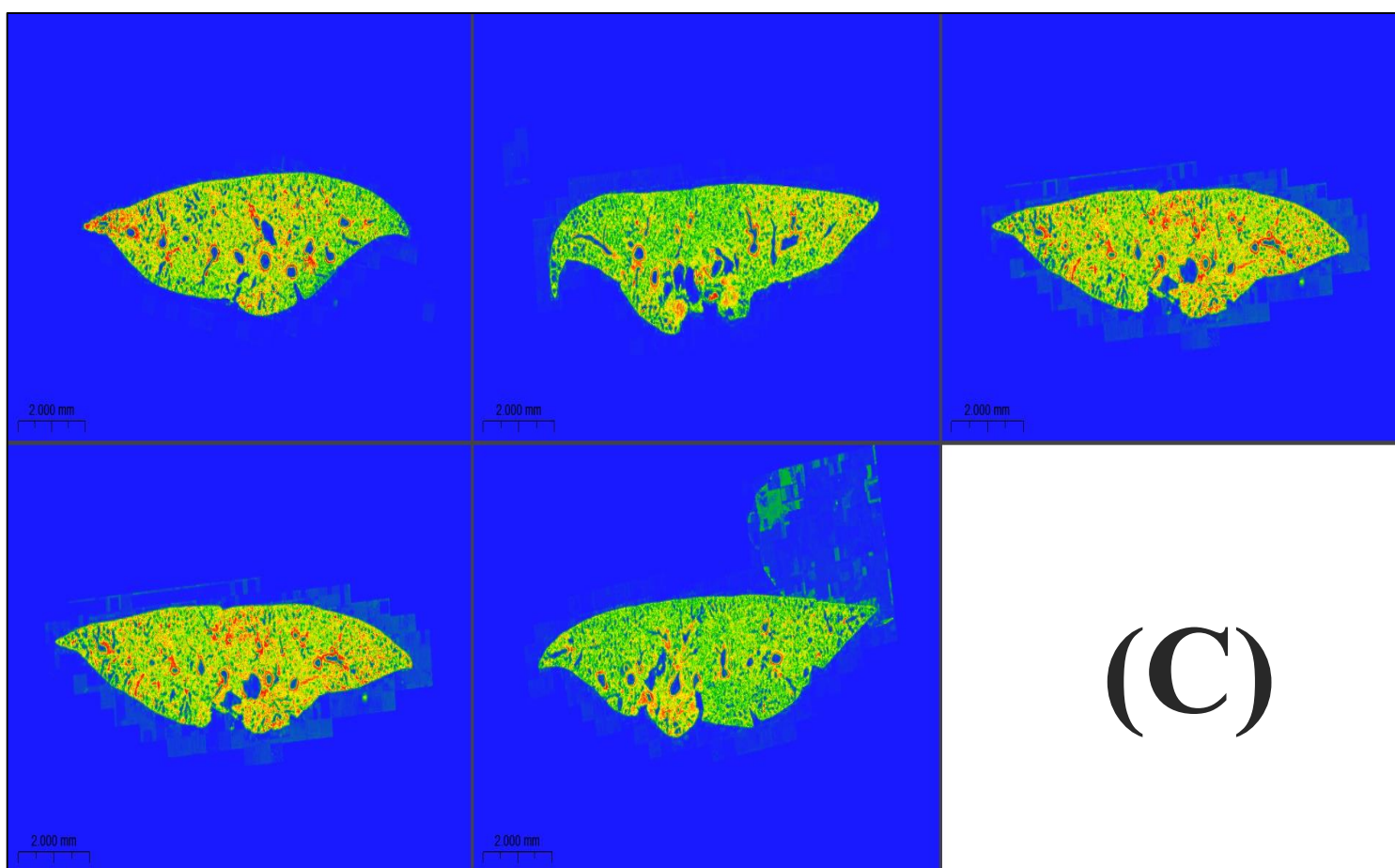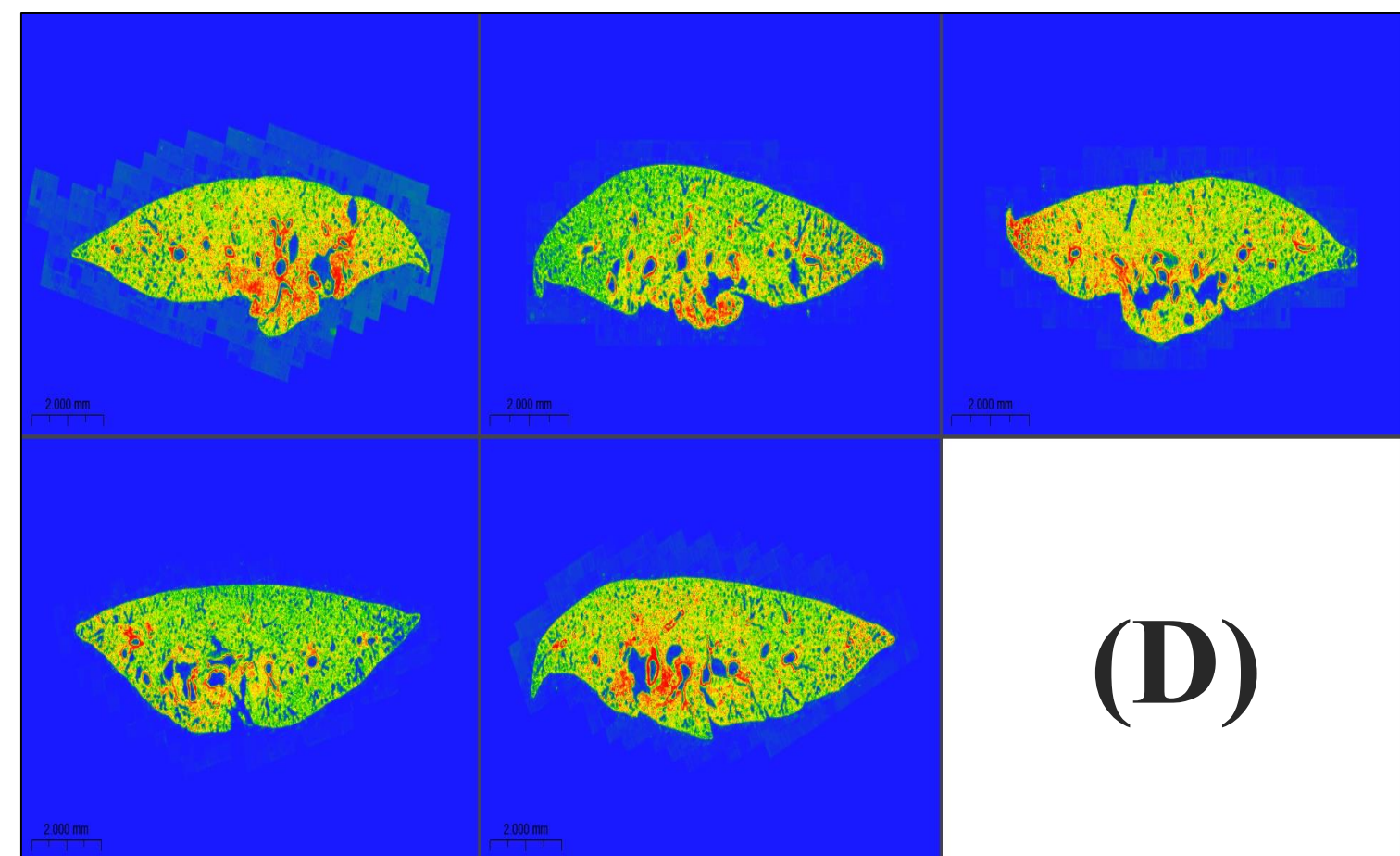

Supplementary Figure 4.

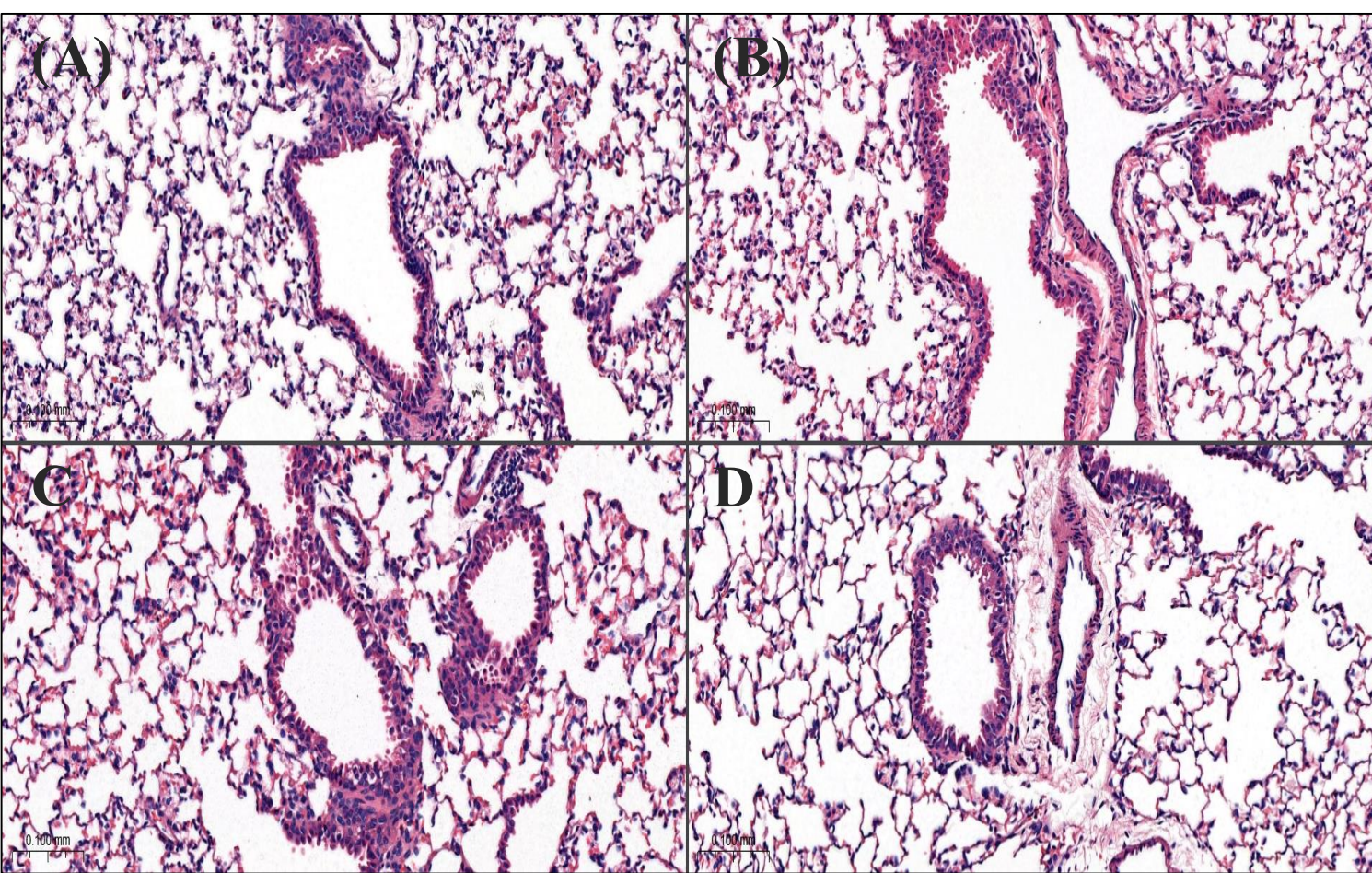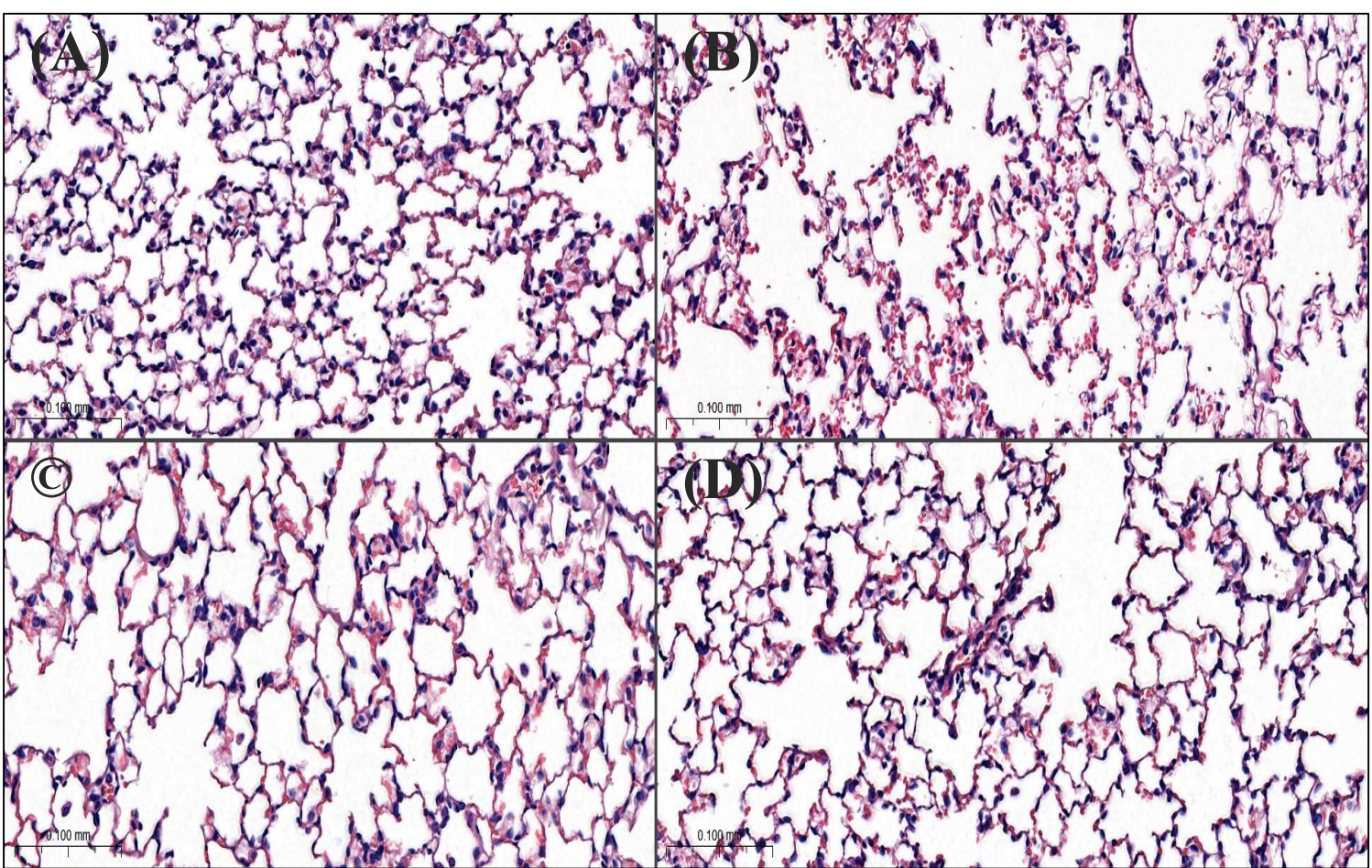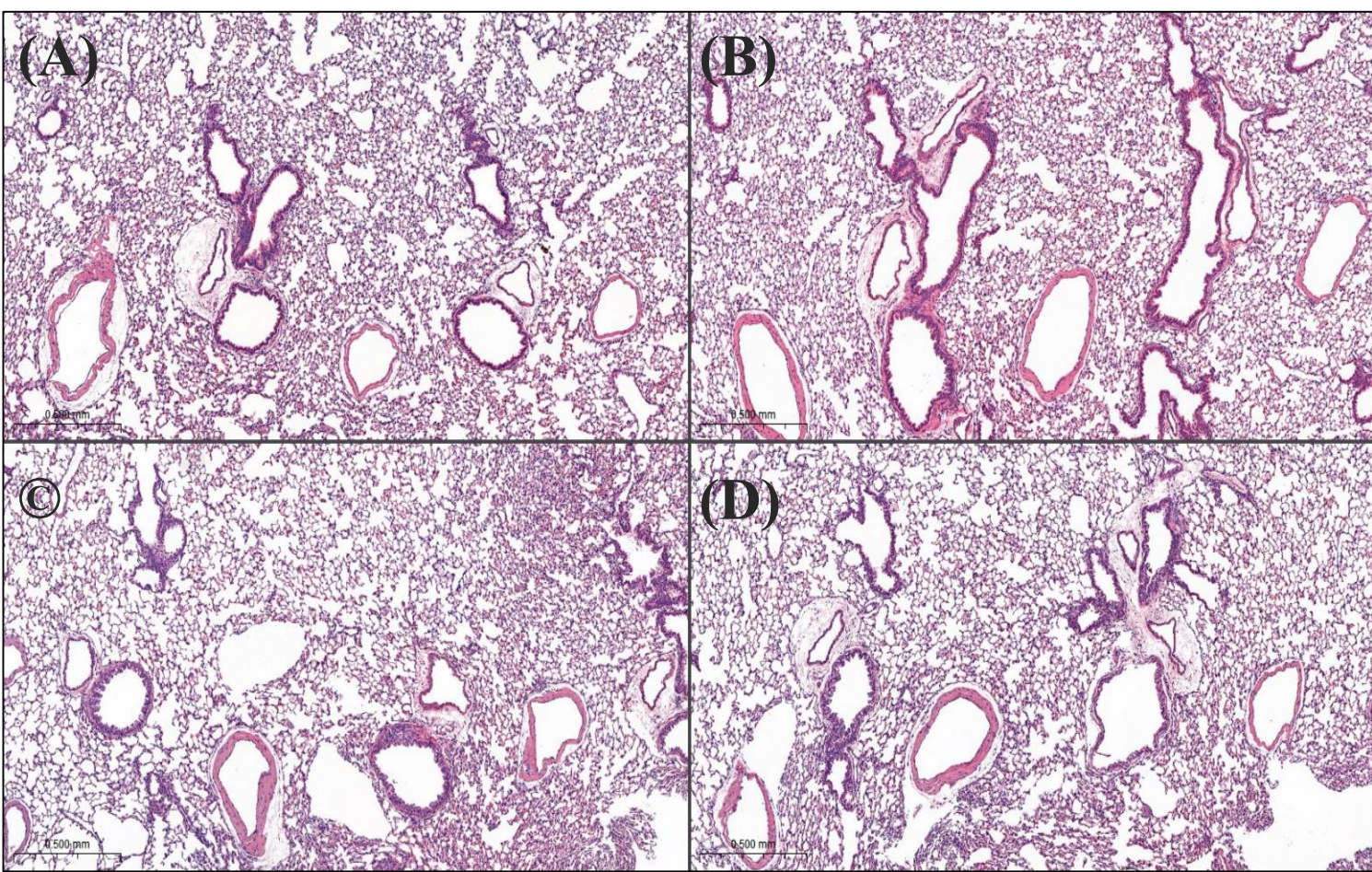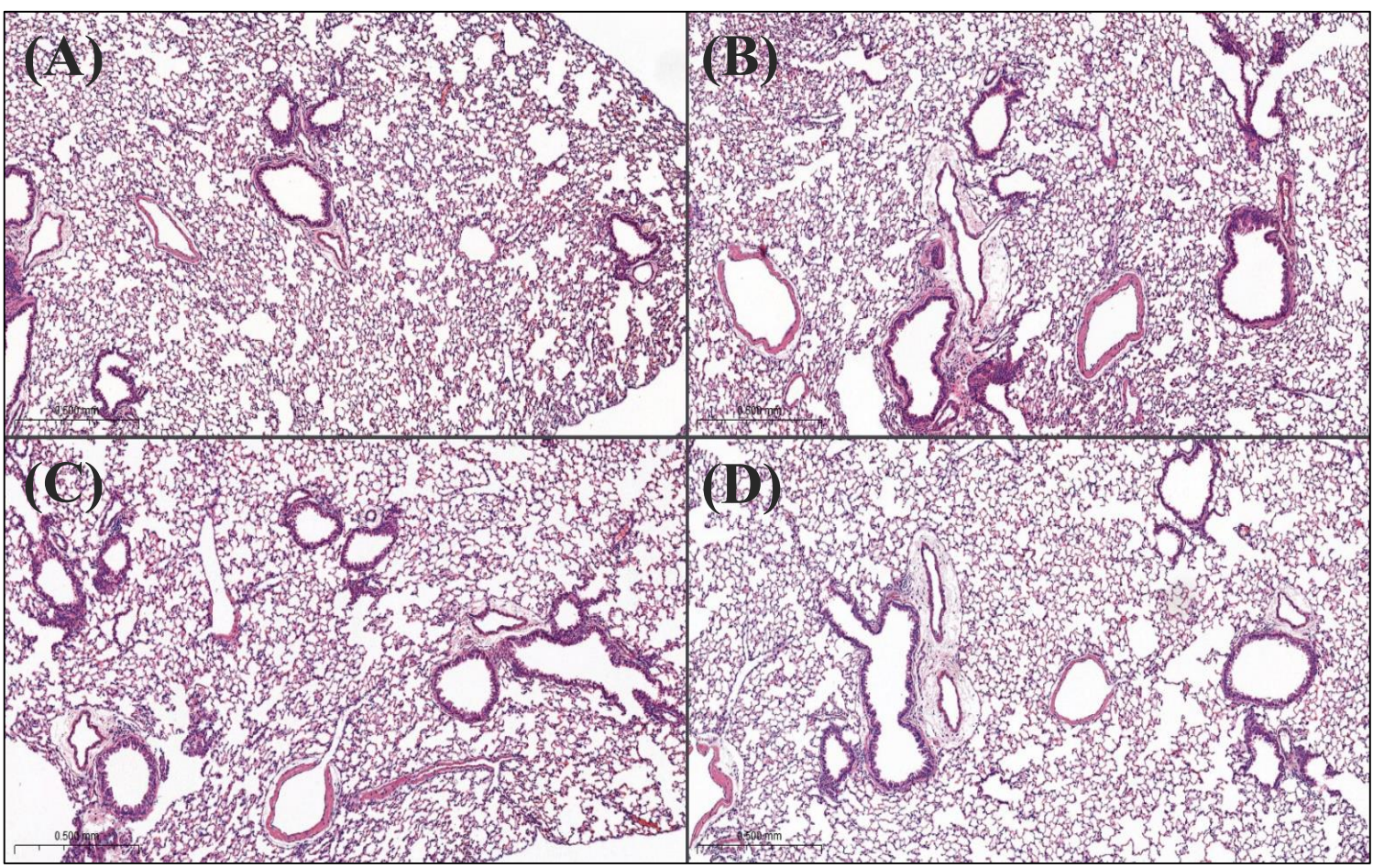

Supplement: dlaf121_Supplementary_Data [file dlaf121_supplementary_data.zip › Supplementary Figure_Acinetobacter phage vB_AbaSt_W16_PK&PD_최종.pdf]
